# Supplementary material for: Needs of key stakeholders to make advance care plans and advance directives for people with dementia: a scoping review
Source: BMC Geriatr. 2025 Nov 10;25:872. doi: 10.1186/s12877-025-06514-3 (PMC12599080; doi:10.1186/s12877-025-06514-3)
Supplement: Supplementary file 2 — Additional file 2: Search string. [file 12877_2025_6514_MOESM2_ESM.pdf]

Search String [UPDATED OCTOBER 2024]

**Database**	**Results**	**Search String**
Pubmed<br>09.04.2024	2,458	(advance directive*[Title/Abstract] OR advance care plan*[Title/Abstract] OR end-of-life decision*[Title/Abstract] OR end-of-life plan*[Title/Abstract] OR decision-making[Title/Abstract] OR patient preference*[Title/Abstract]) AND (dement*[Title/Abstract] OR alzheimer*[Title/Abstract]) AND (2014:2024[pdat])
[Updated]<br>Pubmed<br>31.10.2024	242	(advance directive*[Title/Abstract] OR advance care plan*[Title/Abstract] OR end-of-life decision*[Title/Abstract] OR end-of-life plan*[Title/Abstract] OR decision-making[Title/Abstract] OR patient preference*[Title/Abstract]) AND (dement*[Title/Abstract] OR alzheimer*[Title/Abstract]) AND (2024/4/9:2024/10/31[pdat])
EMBASE<br>09.04.2024	3,305	('advance directive*':ab,ti OR 'advance care plan*':ab,ti OR 'end-of-life decision*':ab,ti OR 'end-of-life plan*':ab,ti OR 'decision making':ab,ti OR 'patient preference*':ab,ti) AND (dement*:ab,ti OR alzheimer*:ab,ti) AND [2014-2024]/py
[Updated]<br>EMBASE<br>31.10.2024	348	('advance directive*':ab,ti OR 'advance care plan*':ab,ti OR 'end-of-life decision*':ab,ti OR 'end-of-life plan*':ab,ti OR 'decision making':ab,ti OR 'patient preference*':ab,ti) AND (dement*:ab,ti OR alzheimer*:ab,ti) AND [2024-2024]/py
SCOPUS<br>09.04.2024	379	(TITLE ("advance directive*" OR "advance care plan*" OR "end-of-life decision*" OR "end-of-life plan*" OR "decision making" OR "patient preference*") AND ABS ("advance directive*" OR "advance care plan*" OR "end-of-life decision*" OR "end-of-life plan*" OR "decision making" OR "patient preference*") AND TITLE (dement* OR alzheimer*) AND ABS (dement* OR alzheimer*)) AND PUBYEAR > 2013 AND PUBYEAR < 2025
[Updated]<br>SCOPUS<br>31.10.2024	42	(TITLE ("advance directive*" OR "advance care plan*" OR "end-of-life decision*" OR "end-of-life plan*" OR "decision making" OR "patient preference*") AND ABS ("advance directive*" OR "advance care plan*" OR "end-of-life decision*" OR "end-of-life plan*" OR "decision making" OR "patient preference*") AND TITLE (dement* OR alzheimer*) AND ABS (dement* OR alzheimer*)) AND PUBYEAR > 2023 AND PUBYEAR < 2025
Cochrane<br>09.04.2024	142	(advance NEXT directive* OR advance NEXT care NEXT plan* OR end-of-life NEXT decision* OR end-of-life NEXT plan* OR decision-making OR patient NEXT preference*):ti OR (advance NEXT directive* OR advance NEXT care NEXT plan* OR end-of-life NEXT decision* OR end-of-life NEXT plan* OR decision-making OR patient NEXT preference*):ab AND (dement* OR alzheimer*):ti OR (dement* OR alzheimer*):ab with Cochrane Library publication date Between Jan 2014 and Mar 2024
[Updated]<br>Cochrane<br>31.10.2024	5	(advance NEXT directive* OR advance NEXT care NEXT plan* OR end-of-life NEXT decision* OR end-of-life NEXT plan* OR decision-making OR patient NEXT preference*):ti OR (advance NEXT directive* OR advance NEXT care NEXT plan* OR end-of-life NEXT decision* OR end-of-life NEXT plan* OR decision-making OR patient NEXT preference*):ab AND (dement* OR alzheimer*):ti OR (dement* OR alzheimer*):ab with Cochrane Library publication date Between Apr 2024 and Oct 2024
